# Oxidative Stress in Muscle Diseases: Current and Future Therapy 2019

**DOI:** 10.1155/2020/6030417

**Published:** 2020-04-16

**Authors:** Andrey Jorge Serra, José Renato Pinto, Marko D. Prokić, Gisela Arsa, Andrea Vasconsuelo

**Affiliations:** ^1^Federal University of Sao Paulo, Sao Paulo, Brazil. Nove de Julho University, Sao Paulo, Brazil; ^2^Florida State University, Tallahassee, USA; ^3^University of Belgrade, Belgrade, Serbia; ^4^Federal University of Mato Grosso, Cuiaba, Brazil; ^5^Universidad Nacional del Sur, Bahia Blanca, Argentina

Increased oxidative stress has important molecular, structural, and functional muscle implications. In pathological conditions, reactive oxygen species (ROS) burst contributes to cellular dysfunction and the progression of muscle diseases. This special issue was designed to advance knowledge in the role of oxidative stress on muscle remodeling, in turn leading to innovative therapeutic approaches in a wide range of muscle diseases. Therefore, this special issue provides recent scientific advancements with researchers and practitioners who work in muscle scope. Articles included in this special issue address the molecular and cellular mechanisms involved in these processes as well as current therapies.

One of the studies published in the special issue examined the impact of 8-week dynamic resistance training with elastic bands on oxidative stress in stroke survivors. Functional fitness, hemodynamic, and cardiac autonomic modulation were also evaluated. The exercise protocol consisted of a sequence of three combinations of two consecutive exercises (i.e., seated row and squat on the chair, vertical chest press and squat on the chair, and knee extension and squat on the chair) in a dynamic manner, without intervals of absolute rest throughout the session. Exercise volume was increased over the 8-week protocol, in which three sets of 6-8 repetitions at moderate intensity (3 to 5 points on Borg scale) were performed in the first four weeks and three sets of 10-12 repetitions at moderate intensity were performed in subsequent weeks. The main findings were a plasma reduction in the levels of thiobarbituric acid reactive substances (TBARS) and carbonyls and an increased activity of superoxide dismutase (SOD). Exercised patients also showed improvements in functional fitness and sympathovagal balance. Therefore, the study has addressed the importance of resistance training using simple equipment such as elastic bands to reduce oxidative stress and improve cardiac autonomic control and functionality in chronic stroke survivors.

Excessive oxidative stress has been stimulated by exercise. The increase in ROS can be dependent on the type, duration, and load of the exercise [1]. ROS at low levels, a phenomenon similar to hormesis, plays an important role in exercise-induced physiological adaptation [2]. However, excessive oxidative stress can result in impaired physical performance and maladaptive skeletal muscle recovery [3]. Recently, experimental studies in rodents have indicated that photobiomodulation (PBM) could be a new alternative to modulate the excessive oxidative stress induced by exercise [4–10]. Several positive findings were highlighted, including (i) lower damage, inflammation, and lipoperoxidation of muscle; (ii) increased activity of antioxidant enzymes; and (iii) delayed muscle fatigue. On a translational perspective of these findings, in this special issue has included a randomized, triple-blind, placebo-controlled crossover trial conducted by S. S. Tomazoni et al. The authors applied PBM (using infrared low-level laser therapy; a total of 850 J of energy) before a high-intensity progressive running test until exhaustion in soccer players. The PBM application brought about improvements in rates of oxygen uptake and time until exhaustion. Moreover, PBM showed to decrease muscle damage, interleukin 6 (IL-6), TBARS, and carbonylated protein levels. On the other hand, PBM led to increased activity of SOD and catalase (CAT) enzymes. Thus, PBM could be a promising approach to improve physical performance and to counter damage, inflammation, and oxidative stress associated with running high-intensity exercise. Further studies are needed to understand the influence of different irradiation dosages and whether the effects published by S. S. Tomazoni et al. can exist in distinct types of exercises.

Several studies support the existence of an interdependent relationship between inflammation and oxidative stress [11, 12]. Among factors playing a key role in skeletal muscle pathophysiology and potentially linking inflammation and oxidative stress, IL-6 is a possible candidate [13]. In this regard, L. Forcina et al. tried to investigate whether elevated circulating levels of the IL-6 could disturb the redox balance in skeletal muscle, independent of tissue damage and inflammatory response. Using NSE/IL-6 transgenic mice characterized by systemically elevated levels of IL-6, the authors show enhanced ROS production and accumulation in the diaphragm muscle. Alteration of the IL-6-linked homeostasis redox appears to require a complex network of interactions that involve the regulation of hydrogen peroxide, nicotinamide adenine dinucleotide phosphate-dependent superoxide scavengention, and mitochondrial antioxidant defense. This special issue also covers the role of ROS played in the development of cell death and cardiovascular diseases. The review article of T. Xu et al. also explored the potential application of the anti-ROS approach in the treatment of cardiovascular diseases. First, the authors documented the different ROS sources and the role of ROS accumulation in vascular dysfunction and cardiac remodeling. Second, the proposal was to address the repercussion of excessive ROS production in the induction of cell death. It has been registered as ROS is closely related to cardiomyocyte apoptosis, autophagy, ferroptosis, and necrosis. Third, the authors reviewed important aspects for several antioxidant agents, including tripeptide glutathione, SOD, CAT, thioredoxin, oxidative stress response transcription factors (e.g., AP-1, HSF1, Nrf2, and FOXO3a), N-acetylcysteine, vitamin E, and NAD^+^. The last concerns were targeted to clinical trials that evaluated the impact of inhibiting oxidative stress in cardiovascular diseases, in which they have failed to mitigate cardiac remodeling and the evolution of heart failure.

Kynurenine (KYN) is a circulating tryptophan metabolite that increases with age and is implicated in several age-related disorders [14]. In this regard, H. Kaiser et al. hypothesized that an increase in KYN with age contributes to muscle atrophy and oxidative stress. *In vitro* experiments showed that KYN treatment of mouse and human myoblasts increased levels of ROS. Young mice had higher muscle lipid peroxidation and reduced muscle size and strength in response to KYN treatment. Aged mice treated with indoleamine 2,3-dioxygenase inhibitor, an enzyme involved in the generation of KYN, showed an increase in muscle fiber size and muscle strength. Protein expression assays revealed very long-chain acyl-CoA dehydrogenase as a factor activated by KYN that may increase ROS and lipid peroxidation. Collectively, these findings make it possible to consider KYN involvement in sarcopenia with age. It appears that the deleterious effects of chronic KYN exposure are mediated by increased oxidative stress. V. Cenni et al. presented an interesting review that describes the effects of the Ankrd2 modulation stimulate by mechanotransduction and cellular ROS. Moreover, they demonstrated a possible relation with the pathogenesis of muscular laminopathies. First, the authors documented the Ankrd2 in striated muscles focusing on expression in skeletal and cardiac muscles, as well as, mechanotransduction in skeletal muscle. They demonstrated how physical exercise could stimulate an Ankrd2 upregulation from oxidative and mechanical stresses. Second, they proposed pathogenic mechanisms in which Ankrd2 might be involved. Third, it showed the perspectives of the study of Lamin-AAnkrd2 interplay, specifically the mechanosignaling through Ankdr2 and therapeutic perspectives. It is important to highlight that the authors showed illustrations for the complex network that involve the relation between Ankrd2 and pathogenesis of muscular laminopathies.

Another interesting study was carried out by S. Q. Rodríguez-Lara et al. which showed a pharmacological approach to reduce ischemia-reperfusion (I/R) damage. This phenomenon can be induced by cellular ROS, resulting in deleterious effects on the lesion, and there is no pharmacological approach to avoid or decrease these dangerous effects. However, the authors presented Telmisartan as a possible pharmacological approach because it seems to affect the concentration and activity of enzymatic scavengers, which could decrease lesion development. For this, male Wistar rats were submitted to treatment with Telmisartan for seven days before I/R lesion and then were evaluated at 1 h, 24 h, 72 h, 7, and 14 days following reperfusion. Muscle samples were obtained to determine SOD-2 and CAT gene expression. The biochemical assay was used to determine the oxidative and antioxidative markers. Histological tissue evaluation from the right gastrocnemius muscle was performed. The main results were that the approach of Telmisartan (i) produced changes in the SOD-2 and CAT gene expression regarding reperfusion, (ii) reduced oxidative markers levels in the local tissue, and (iii) promoted injury attenuation between 24 h and 14 days. A novel pharmacological approach with Telmisartan demonstrated to be promissory to reduce damages during I/R damage. The elderly population has been increasing, and sarcopenia became a vital topic to be studied because it affects autonomy in daily life and compromises the quality of life [15]. In this issue, N. Z. Azlan et al. described several strategies to counter sarcopenia and among them presented the *Chlorella vulgaris*, a green alga that seems to promote muscle regeneration. The authors aimed to establish the effects of the differentiation of myoblast cells during the formation of mature myotubes in culture. For this, human myoblast cells were obtained from young men and women and then were cultured on adequate conditions and stimulates until reaching senescence. Cells were treated with *Chlorella vulgaris* and incubated for up to seven days to induce differentiation. Different techniques were used to analyze the ability of *Chlorella vulgaris* to promote myoblast differentiation (e.g., cellular morphology, real-time monitoring, cell proliferation, senescence-associated B-galactosidase expression, myogenic differentiation, myogenic expression, and cell cycle profiling). *Chlorella vulgaris* improved the regenerative capacity of young and senescent myoblasts, stimulating differentiation, demonstrating to have a high potential to treat sarcopenia.

K. P. Dzik et al. show in this issue the relations among muscle atrophy and vitamin D deficiency. The authors describe the muscle function affected by vitamin D deficiency and the development of muscle atrophy. Lumbar back pain (LBP) patients showed paravertebral muscle atrophy. The authors investigated muscle atrophy markers, signaling proteins, and mitochondrial capacity in LBP patients according to sex and vitamin D levels. Men and women were distributed into three groups according to levels of vitamin D received for five weeks. Three dosages of vitamin D were established to reach deficiency, normal, and above normal levels. The key results were that the vitamin D deficiency-induced stress oxidative, which is involved in a cascade of enzymatic events that leads to muscle atrophy and mitochondrial dysfunction, is most present in women due to higher Atrogin-1 levels. Normal or higher levels of vitamin D increase the mitochondrial function and inhibit muscle atrophy.

C. D. Pandya et al. described the role of oxidative stress on aging and their mechanisms that results in sarcopenia and osteoporosis. They empathize the possible effects of regulation of arginase from oxidative stress on the survival and differentiation of myoblasts. The authors aimed to investigate the arginase activity and expression in the skeletal muscle in young and aged mice. First, arginase activity and arginase 1 expression were determined. Second, the expression of oxidative stress-related signaling molecules in muscles of aged mice was performed. Third, *in vitro* studies were conducted with myoblast cell line (C2C12) and arginase inhibitor (ABH). The main results were an elevated arginase activity in aged muscle and a reduced NO production that occurs by the competition for L-arginine and eNOS uncoupling. Also, the authors highlighted that it is possible to prevent or slow down the degenerative effect in muscle aging by limiting arginase activity.

We hope that this special issue has been successful in providing new insights into the impact of oxidative stress in muscle physiology and several muscle disorders. Editors considered the interdisciplinary nature of the papers included as necessary to expand on fundamental concepts, i.e., basic research as well as those important to applied sciences. Therefore, the readers of this special issue should find information of interest, relevant to their respective areas of scientific investigations.
